# An Eco-Friendly Acid Leaching Strategy for Dealkalization of Red Mud by Controlling Phase Transformation

**DOI:** 10.3390/ma15020580

**Published:** 2022-01-13

**Authors:** Jiaming Wu, Tianyu Lei, Beibei Wang, Shuwei Ma, Yulong Lin, Xiaolei Lu, Zhengmao Ye

**Affiliations:** 1 School of Materials Science & Engineering, University of Jinan, Jinan 250022, China; w1324491461@163.com (B.W.); linlin001177@163.com (Y.L.); 2 Shandong Academy of Building Research Co., Ltd., Jinan 250031, China; lty121133@163.com; 3 Shandong Institute for Product Quality Inspection, Jinan 250199, China; ma_shuwei@yeah.net; 4 Shandong Provincial Key Laboratory of Preparation and Measurement of Building Materials, Jinan 250022, China; mse_luxl@ujn.edu.cn

**Keywords:** red mud, phase transformation, acid leaching, resource utilization, nonsecondary pollution

## Abstract

The alkaline components in red mud represent one of the crucial factors restricting its application, especially for the construction and building industry. The phase state of alkaline components has a significant influence on the dealkalization of red mud. In this work, an environmentally friendly acid leaching strategy is proposed by controlling the phase transformation of red mud during active roasting pretreatment. With a moderate roasting temperature, the alkaline component is prevented from converting into insoluble phases. After acid leaching with a low concentration of 0.1 M, a high dealkalization rate of 92.8% is obtained. Besides, the leachate is neutral (pH = 7) and the valuable metals in red mud are well preserved, manifesting a high selectivity and efficiency of diluted acid leaching. The calcination experiment further confirms the practicability of the strategy in the construction field, where the cementitious minerals can be formed in large quantities. Compared with the traditional acid leaching routes, the diluted acid leaching strategy in this work is acid saving with low valuable element consumption. Meanwhile, the secondary pollution issue can be alleviated. Hence, the findings in this work provide a feasible approach for the separation and recovery of alkali and resource utilization of red mud.

## 1. Introduction

**Figure 1:**
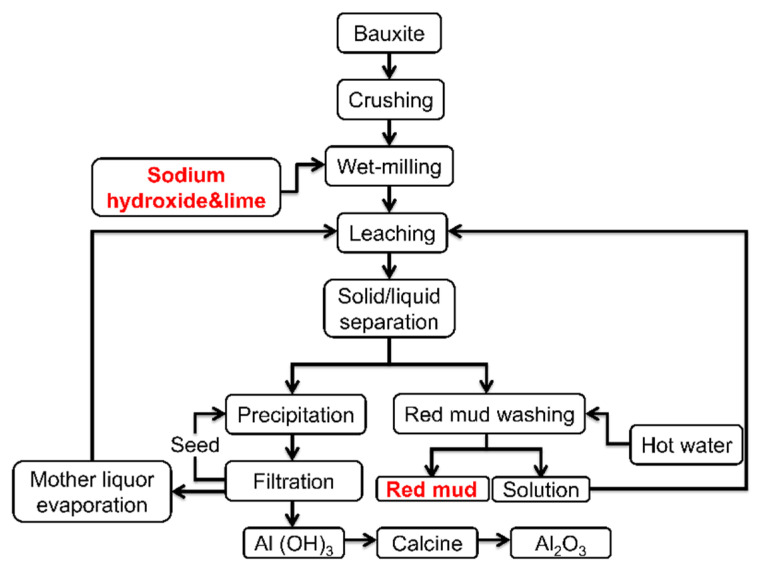
The flow chart of the Bayer process [20].

Red mud is the industrial waste discharged from the alumina preparation with bauxite [1,2]. Generally, extracting 1 t of alumina would produce about 0.5–2.0 t of red mud [3,4,5]. According to the statistical data, more than 160 million t of red mud are produced in the world every year [6,7,8]. Red mud not only occupies vast areas of agricultural land but also causes a certain degree of environmental pollution on soil and groundwater [9,10,11]. Thus, it is urgent to develop the effective utilization and disposal of red mud. In the Bayer process (Figure 1), bauxite is treated with sodium hydroxide solution in a pressure vessel, leading to the high alkalinity of red mud [12,13,14]. Unfortunately, high alkalinity is a critical factor limiting the use of red mud, such as in the iron industry, construction industry, catalysts, energy storage, and wastewater treatment [15,16,17]. As for the construction industry, high alkalinity has adverse effects on clinker sintering and the relevant service performance, leading to alkali efflorescence, low strength, and inadequate durability [18,19]. Thus, the dealkalization of red mud is beneficial to promoting the recycling of red mud and reducing environmental problems.

Many researchers have investigated the removal of the alkali compounds in red mud. The commonly used techniques for dealkalization mainly include water leaching, seawater neutralization, salt precipitation, hydrothermal calcification, acid leaching, and acid gas neutralization [21,22,23,24]. Water leaching is a simple method for dealkalization, whereas it is not efficient at the removal of insoluble alkali in red mud. Salt precipitation is another strategy based on an ion substitution mechanism, including seawater neutralization, salt precipitation, and hydrothermal calcification. In this route, the alkaline component is transformed into a more insoluble form rather than being destroyed and eliminated. Thus, the proportion of the alkaline component in red mud is not decreased, and it is inevitable to introduce new components in the product. The acid neutralization strategy is an effective way to remove the alkaline component from red mud, especially for insoluble alkali. Acid leaching is a simple and feasible method, without needing complicated equipment and harsh conditions in the acid gas neutralization process. The alkaline component in red mud can be successfully neutralized and leached by various acids, such as mineral acid, organic acid, waste acid, and bio acid. Hu et al. reported that 94.70% of Na was removed by using sulfuric acid leaching with a concentration of 1.6 M [25]. Wang et al. neutralized red mud with bio acid extracted from fallen leaves, and the pH value of red mud was reduced from 9.88 to 6.65 after treatment [21]. However, a high dealkalization efficiency always requires a high acid concentration, which results in the dissolution of other valuable metals in red mud, and the waste acid causes secondary pollution [20,26,27]. Therefore, development and improvement of the acid leaching method are still required.

Red mud possesses the characteristics of a complex structure with frame holes, large specific surface area, and high water content [28]. Tiny particles are always connected to form agglomerate with high adhesion, which is considered an obstacle to the treatment and utilization of red mud. Interestingly, it is reported that active roasting is conducive to breaking the connection between particles and loosening the frame structure of red mud [29]. Additionally, the relevant phase transformation and chemical reaction during active roasting are beneficial to increasing the dealkalization rate. Liu et al. found the insoluble alkaline phases could be transformed into dissoluble phases with 850 °C calcination. The dealkalization rate was 97.0% after four-stage water leaching [30]. Gong et al. removed 97.6% alkali through acid leaching after a 500 °C pretreatment was employed [31]. Based on this, if controlling the phase transformation of red mud during active roasting, especially for alkaline components, the acid leaching conditions may be optimized.

In this work, a diluted acid leaching method was proposed, with the combination of active roasting pretreatment. By controlling the phase transformation, especially the alkali component, acid leaching with high selectivity and efficiency could be realized. With utilization of the diluted acid leaching, the secondary pollution could be highly alleviated, and it also benefited the valuable metals’ recovery of red mud. Compared with the raw red mud, more cementitious minerals were formed in the calcination product when using dealkalization red mud as the raw material. The diluted acid leaching strategy in this work may lay a foundation for eco-friendly dealkalization and red mud utilization. Additionally, it also provides a successful practice for red mud according to the phase transformation controlling conception.

## 2. Materials and Methods

### 2.1. Red Mud

**Figure 2:**
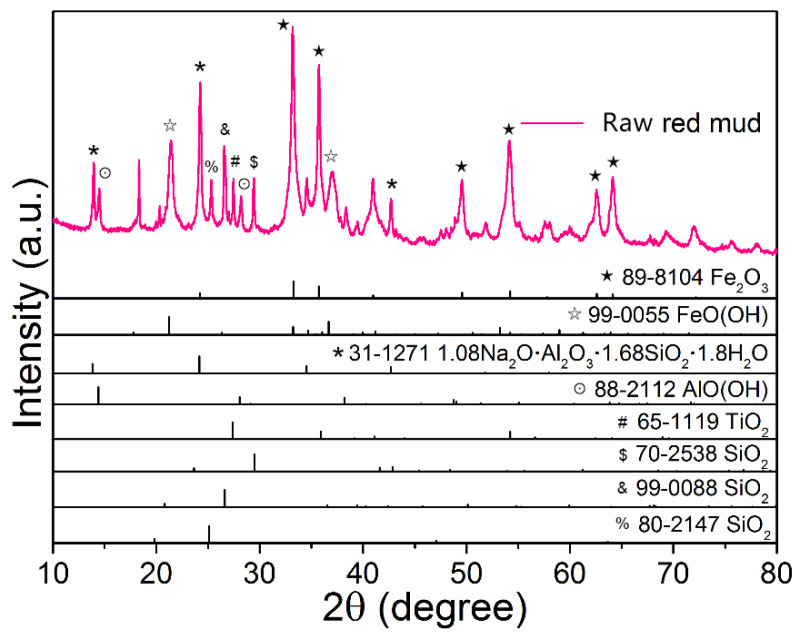
XRD pattern of the raw red mud.

Red mud was collected by Bayer process aluminum from a factory in Shandong Province (China). The mineral and chemical composition of the raw red mud were characterized (Figure 2 and Table 1). As shown in Table 1, raw red mud mainly consisted of Fe_2_O_3_, Al_2_O_3_, SiO_2_, Na_2_O, TiO_2_, and CaO. As shown in Figure 2, the main minerals in raw red mud were hematite (Fe_2_O_3_), boehmite (AlO(OH)), goethite (FeO(OH)), and hydrated sodium aluminosilicates (1.08Na_2_O·Al_2_O_3_·1.68SiO_2_·1.8H_2_O). In addition, a small amount of quartz phase (SiO_2_) and rutile phase (TiO_2_) were presented in the raw red mud.

### 2.2. Sample Preparation

Firstly, the raw red mud was crushed with a particle size below 50 μm, and dried at 105 °C for 6 h to remove free water. Then, the dried red mud was subjected to thermal analysis to determine the temperature range of the active roasting process. Based on the analysis results, red mud was calcined in a muffle furnace with a temperature range of 200–1000 °C and a temperature gradient of 100 °C. The heating rate was 4 °C/min and the calcining process was kept for 4 h under the air atmosphere. After furnace cooling to room temperature, the calcined samples were ground and named after the calcined temperature, which is RM-200, RM-300, RM-400, RM-500, RM-600, RM-700, RM-800, RM-900, and RM-1000, respectively. According to the phase transformation of red mud, the calcined samples RM-800 and RM-500 were selected for acid leaching with the liquid-solid ratio of 30:1. The concentration of the hydrochloric acid solution was 0.1, 0.25, 0.5, and 1 M, respectively. After leaching at room temperature for 5 h, the precipitate was separated from leachate by centrifugation. The leachate pH was analyzed. The leached red mud powder was washed by water three times and named after acid concentrations, such as RM-500A (0.1 M), RM-800A0.1, RM-800A0.25, RM-800A0.5, and RM-800A1, respectively. In addition, a control sample was constructed, where RM-500 was treated by water leaching and named RM-500W.

**Figure 3:**
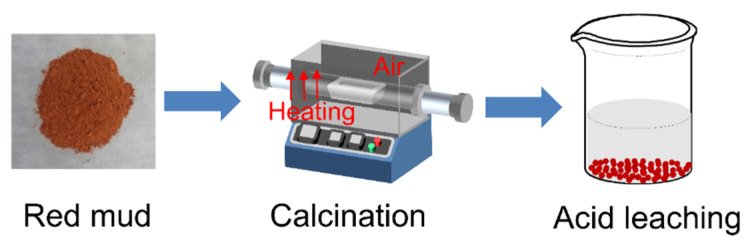
The schematic diagram for the diluted acid leaching strategy of red mud.

The scheme of the diluted acid leaching process is shown in Figure 3. For the calcination experiment, the dealkalization red mud RM-500A was sintered at 1350 °C for 2 h after being mixed with limestone. Raw red mud was also sintered in the same condition for comparison. The raw materials used in this work were purchased from Sinopharm Chemical Reagent Co., Ltd. (Shanghai, China).

### 2.3. Characterization

X-ray diffraction (XRD) patterns were measured to analyze the crystalline phases of samples by using an X-ray powder diffractometer (Rigaku Ultima IV, Rigaku Corporation, Tokyo, Japan). X-ray fluorescence (XRF) analysis was used to determine the composition of red mud samples (S8 TIGER 2, Bruker, Billerica, MA, USA). The morphology and the atomic distribution of samples were performed on a scanning electron microscope (SEM, FEI-QUANTA-FEG 250, Thermo Fisher Scientific, Waltham, Massachusetts, USA) equipped with an energy-dispersive spectrometer (EDS, Oxford INCA Energy X-MAX-50, Oxford Instruments, Oxford, England). For SEM measurement, the sample powder was dispersed on the carbon conductive adhesive. The selected accelerating voltage was 10 KeV and the aperture hole was 2.5. The thermal phase transformation behavior of the red mud sample was evaluated by thermogravimetric analysis (TG) with a Mettler Toledo TGA/DSC thermal analyzer (METTLER TOLEDO, Zurich, Swiss). Because the phase transformation of red mud has nothing to do with the heating rate and atmosphere, the TG test was conducted under an Ar atmosphere with a heating rate of 10 °C/min, and the recorded temperature range was from 30 to 1000 °C.

## 3. Results and Discussion

### 3.1. Thermal Phase Transformation Behavior of Red Mud

**Figure 4:**
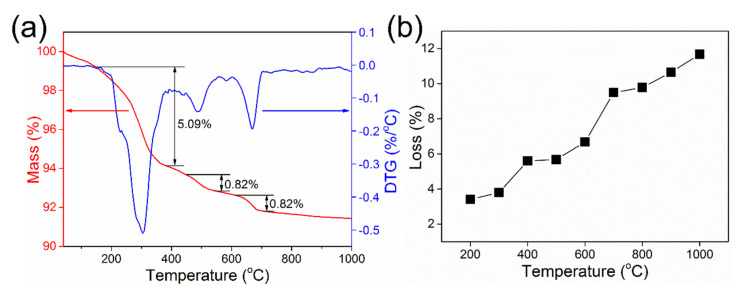
(**a**) TG-DTG curves of the red mud sample, (**b**) mass loss of calcined samples with different calcination temperatures.

Due to the dehydration and decomposition of minerals at high temperatures, red mud usually undergoes phase transformation during the active roasting process. The relevant temperature ranges were revealed by the TG test. As shown in Figure 4a, when the test temperature is increased from 30 to 1000 °C, three stages of mass loss appear in the TG curve. The first stage of mass loss is presented at 200–400 °C, and the second mass loss is observed at approximately 450–500 °C. The mass loss at below 500 °C can be attributed to the dehydration of the chemically adsorbed water in hydrous minerals, such as FeO(OH) and AlO(OH). The third mass loss at 600–700 °C may be ascribed to the dehydration and phase transformation of 1.08Na_2_O·Al_2_O_3_·1.68SiO_2_·1.8H_2_O, which needs to be clarified by XRD analysis. Then, the mass loss of calcined samples from RM-200 to RM-1000 was also recorded, which was consistent with the TG analysis (Figure 4b).

**Figure 5:**
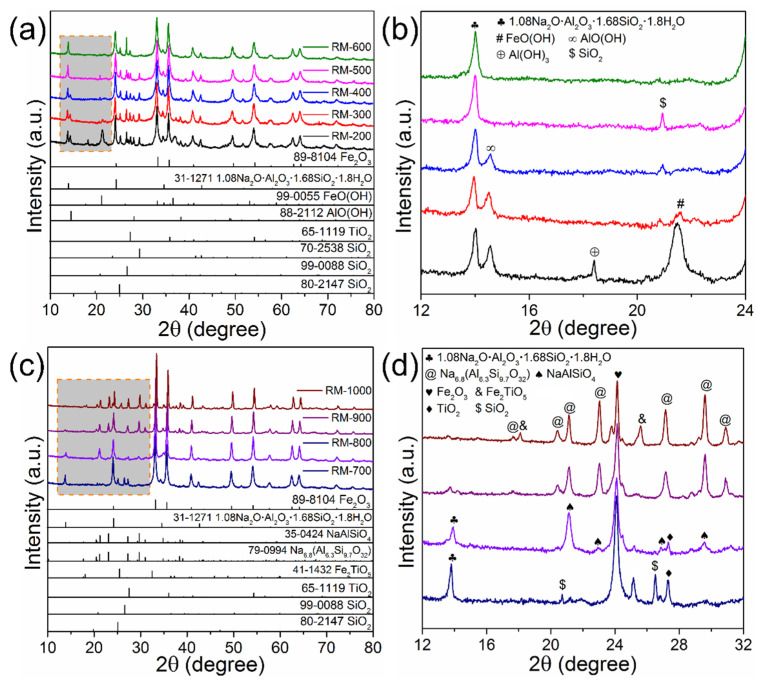
XRD patterns of red mud after calcination (**a, b**) RM-200, RM-300, RM-400, RM-500, RM-600, (**c, d**) RM-700, RM-800, RM-900, RM-1000.

To investigate the phase transformation in red mud during the calcination process, the XRD patterns of RM-200 to RM-1000 were measured (Figure 5). When the calcination temperature is increased from 200 to 400 °C, the diffraction peaks at 21.2°, 33.2°, and 36.7° ascribed to FeO(OH) are sharply decreased. According to Equation (1), FeO(OH) is dehydrated and converted into Fe_2_O_3_ [32]. The dehydration of FeO(OH) corresponds to the first mass loss stage in the TG analysis. When the calcination temperature is increased to 500 °C, the diffraction peaks of AlO(OH) at 14.5°, 28.1°, and 38.3° disappear. As shown in Equation (2), AlO(OH) would lose the crystal water in the dehydration process [33]. However, there is no diffraction peak of Al_2_O_3_ in the XRD patterns, which may be ascribed to the amorphous form of the dehydrated product. A similar phenomenon was observed in previous reports [8,34]. As for the alkaline component, the diffraction peaks of 1.08Na_2_O·Al_2_O_3_·1.68SiO_2_·1.8H_2_O weakened when the temperature reached 700 °C, which matches well with the third mass loss stage of red mud. As the calcination temperature further increases to 800 °C, the peak intensity of 1.08Na_2_O·Al_2_O_3_·1.68SiO_2_·1.8H_2_O is significantly decreased, while the diffraction peaks of NaAlSiO_4_ and Na_6.8_(Al_6.3_Si_9.7_O_32_) appear. Therefore, apart from the dehydration of 1.08Na_2_O·Al_2_O_3_·1.68SiO_2_·1.8H_2_O, there is also a phase transformation of the sodium aluminosilicate mineral. It is noted that the Si/Al ratio of sodium aluminosilicates is increased with the calcination temperature, and the diffraction peaks of SiO_2_ almost vanish at 800 °C. Therefore, the main reason accounting for the phase transformation is the combination of the active component (e.g., SiO_2_ and Al_2_O_3_) and sodium aluminosilicates at high temperatures [35]. The relevant phase transformation mechanism can be illustrated by Equation (3–5). Additionally, as the calcination temperature increased to 1000 °C, Fe_2_TiO_5_ is formed by the combination of Fe_2_O_3_ and TiO_2_ (Equation (6)) [36]:

2 FeO(OH) → Fe_2_O_3_ + H_2_O (1)

2 AlO(OH) → Al_2_O_3_ + H_2_O (2)

1.08Na_2_O·Al_2_O_3_·1.68SiO_2_·1.8H_2_O + 0.48 SiO_2_ + 0.08 Al_2_O_3_ → 2.16 NaAlSiO_4_ + 1.8 H_2_O (3)

3.15 (1.08Na_2_O·Al_2_O_3_·1.68SiO_2_·1.8H_2_O) + 4.41 SiO_2_ → Na_6.8_(Al_6.3_Si_9.7_O_32_) + 5.67 H_2_O (4)

6.3 NaAlSiO_4_ + 3.4 SiO_2_ + 0.25 Na_2_O → Na_6.8_(Al_6.3_Si_9.7_O_32_) (5)

Fe_2_O_3_ + TiO_2_ → Fe_2_TiO_5_ (6)

### 3.2. The Diluted Acid Leaching of Red Mud

Based on the above analysis, the crystalline phases in red mud vary with calcination temperature, especially for the alkali component. For example, after calcination at 500 °C, sodium aluminosilicates maintain the raw 1.08Na_2_O·Al_2_O_3_·1.68SiO_2_·1.8H_2_O phase. When the calcination temperature is above 800 °C, the combination with active SiO_2_ and Al_2_O_3_ causes the phase transformation of sodium aluminosilicates, where 1.08Na_2_O·Al_2_O_3_·1.68SiO_2_·1.8H_2_O is converted to the high Si/Al ratio phases, such as NaAlSiO_4_ and Na_6.8_(Al_6.3_Si_9.7_O_32_). To illustrate the dealkalization and selectivity of acid leaching for different alkali phases, the RM-800 sample containing two kinds of sodium aluminosilicates was selected for the acid leaching test.

**Figure 6:**
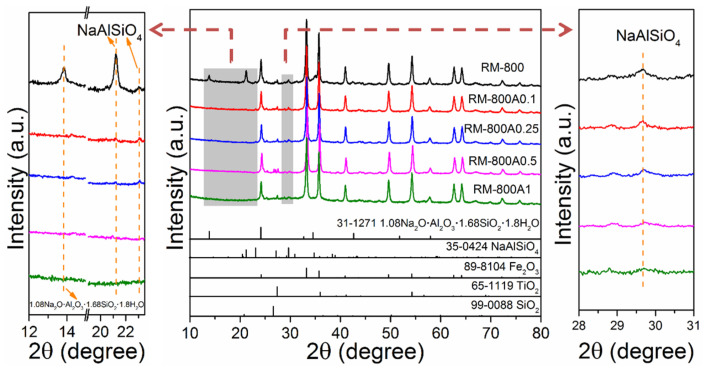
XRD patterns of the RM-800 sample after acid leaching with different concentrations.

**Figure 7:**
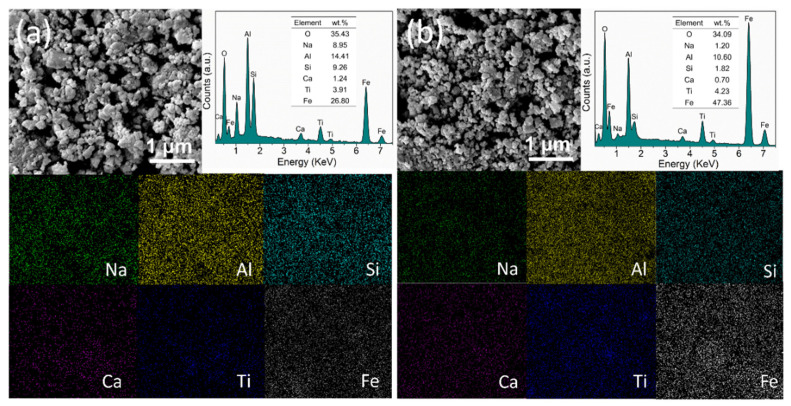
SEM-EDS analysis of RM-800 (**a**) and RM-800A0.1 (**b**).

After leaching with different acid concentrations, the crystalline phases of RM-800 leached samples were determined by XRD patterns (Figure 6). Compared to RM-800, the diffraction peak at 13.9° attributed to 1.08Na_2_O·Al_2_O_3_·1.68SiO_2_·1.8H_2_O disappears in all acid leached samples, indicating 1.08Na_2_O·Al_2_O_3_·1.68SiO_2_·1.8H_2_O can be completely removed with a low acid concentration. As for NaAlSiO_4_, although the diffraction peak at 21.3° is significantly decreased in RM-800A0.1, the existence of peaks at 23.1° and 29.8° in the RM-800A0.1, RM-800A0.25, and RM-800A0.5 samples indicates the high Si/Al ratio phase NaAlSiO_4_ remains in red mud even after strong acid leaching. The result can also be illustrated by SEM-EDS semi-quantitative analysis. As shown in Figure 7, the particle size of the RM-800 sample is decreased after acid leaching, and the content of the Na is reduced from 8.95 wt.% of RM-800 to 1.20 wt.% RM-800A0.1. The results indicate that sodium aluminosilicates are less acid soluble after phase transformation, which is mainly attributed to the increase of the corrosion resistance with a high Si/Al ratio of clay materials [37]. As shown in the XRD pattern, NaAlSiO4 is almost removed until the acid concentration increased to 1 M. However, in this case, the pH of leachate is reduced to 1, and the mass loss of the red mud sample is increased to 37.97 wt.% (Table 2). Acidic leachate is bound to secondary pollution, and the excessive acid dosage may also lead to the loss of valuable metals in red mud. Therefore, it is necessary to control the phase transformation of alkali compounds in red mud for adapting the appropriate and efficient acid leaching condition to achieve a high dealkalization rate and low-pollution waste.

**Figure 8:**
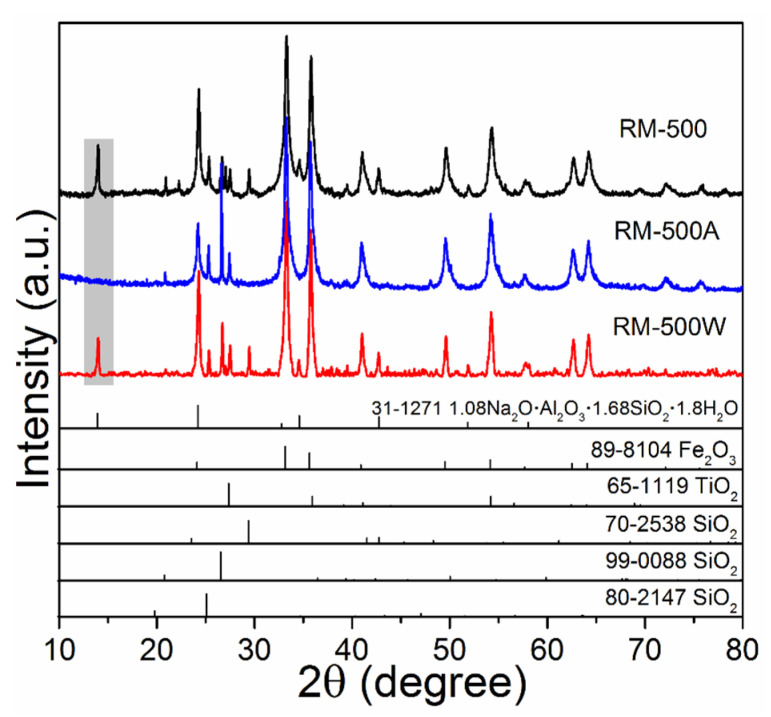
XRD patterns of the RM-500 sample after acid leaching (0.1 M) and water leaching.

After investigating the dealkalization behavior of different alkali phases, it is found that 1.08Na_2_O·Al_2_O_3_·1.68SiO_2_·1.8H_2_O is suitable for acid leaching with a low concentration. Thus, RM-500 with all alkaline components of 1.08Na_2_O·Al_2_O_3_·1.68SiO_2_·1.8H_2_O was used for dilute acid leaching. As presented in Figure 8, the diffraction peaks of 1.08Na_2_O·Al_2_O_3_·1.68SiO_2_·1.8H_2_O in the RM-500A0.1 sample completely disappear, indicating the effectiveness of dealkalization with 0.1 M acid solution. Moreover, the leachate is neutral (pH = 7), which solves the secondary pollution problem faced by traditional acid leaching. The dealkalization mechanism is further illustrated by water leaching. The related diffraction peaks are decreased when compared RM-500W to RM-500, implying 1.08Na_2_O·Al_2_O_3_·1.68SiO_2_·1.8H_2_O can be partially dissolved in water and be removed. The dehydration of hydrous minerals FeO(OH) and AlO(OH) in the RM-500 sample is also conducive to breaking the connection between red mud particles, which facilitates the acid leaching process.

**Figure 9:**
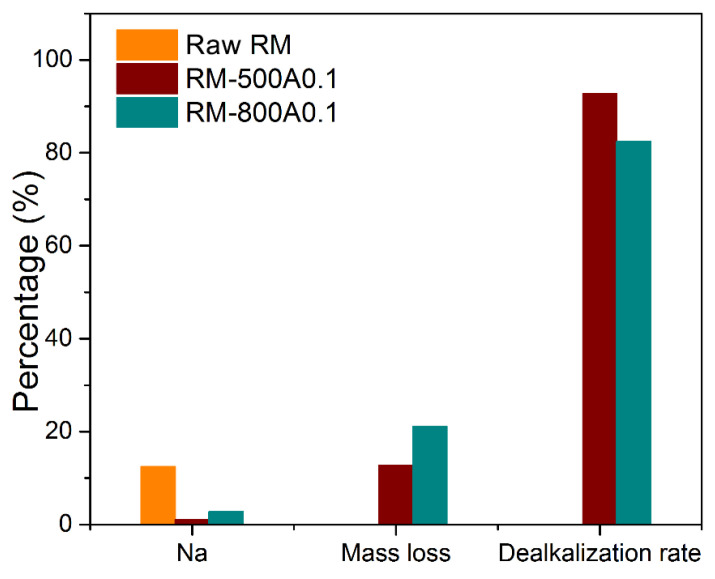
The Na content, mass loss, and dealkalization rate of three samples.

The dealkalization rate of red mud was evaluated by the chemical composition of samples characterized by XRF. As shown in Figure 9, the weight loss and Na content are the lowest, and the alkali removal rate is the highest in RM-500A. In Table 3, the content of Na is 1.02 wt.% and 2.78 wt.% in RM-500A and RM-800A0.1, respectively. The relevant dealkalization rate was determined according to Equation (7) [38], where X is the dealkalization rate; C_0_ and C_1_ are the content of Na of the red mud sample before and after acid leaching; and m_0_ and m_1_ are the weight of the red mud sample before and after acid leaching, respectively. The dealkalization rates are 92.8% and 82.4% for RM-500A and RM-800A0.1, respectively. By the exploration of phase transformation in red mud during the active roasting pretreatment, the more efficient dealkalization of RM-500 than RM-800 benefits from the controlling of the sodium aluminosilicates phase. In addition, the contents of valuable metals, including Fe, Al, and Ti, are increased after diluted acid leaching. The valuable metals can be well preserved in red mud by the advantage of such a low acid concentration, which is mainly ascribed to the high selectivity of acid leaching to alkali in calcined red mud:

(7) $$X=\frac{m_{0}C_{0}{-m}_{1}C_{1}}{m_{0}C_{0}}\;\text{×}\;100\%$$

**Figure 10:**
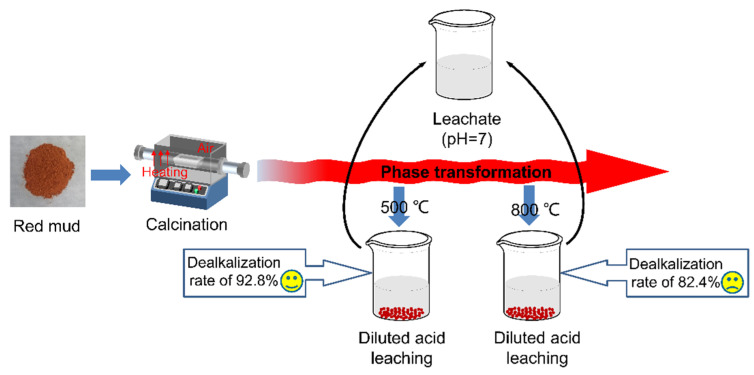
Schematic diagram of the diluted acid leaching strategy.

The schematic diagram of the diluted acid leaching strategy is shown in Figure 10. By controlling the phase transformation in red mud during heat treatment, a more environmentally friendly acid leaching strategy can be achieved. As listed in Table 4, the high dealkalization rate obtained by the diluted acid leaching route in this work is comparable to those reported in the literature [29,30,31,38,39,40]. Compared to traditional acid leaching, the diluted acid leaching strategy is in favor of saving acid usage, reducing valuable element consumption, and alleviating secondary pollution. Different from the commonly used active roasting method with water leaching, the use of acid solution not only reduces the buffer time but also could remove the alkali without multiple leaching, which is more effective and economical. The advantages of diluted acid leaching in this work originate from the control of phase transformation in red mud during heat treatment, and it has potential application in various fields, such as cement, storage, and catalyst.

**Figure 11:**
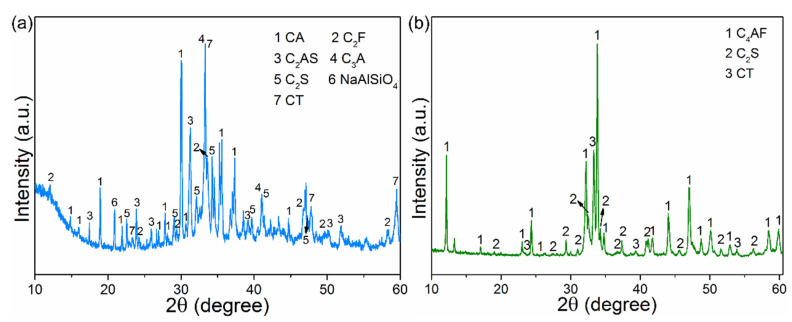
XRD patterns of the sintered samples: the raw red mud (**a**), and the dealkalization red mud (**b**).

To illustrate the practicability of the diluted acid leaching strategy in the construction field, the calcination experiment was implemented. The addition of limestone was determined by the mineral composition of the calcined product. For example, according to the XRF data of the dealkalization red mud RM-500A, 4CaO·Al_2_O_3_·Fe_2_O_3_ (C_4_AF), 2CaO·SiO_2_ (C_2_S), and CaO·TiO_2_ (CT) were expected to be formed in the calcined product. Thus, limestone was added with an adequate stoichiometric ratio of CaO to other metal oxides. As shown in Figure 11a, after calcination of red mud and limestone, cementitious minerals, including CaO·Al_2_O_3_ (CA), C_2_S, 3CaO·Al_2_O_3_ (C_3_A), and 2CaO·Fe_2_O_3_ (C_2_F), are formed, accompanying a large number of inert minerals, such as 2CaO·Al_2_O_3_·SiO_2_ (C_2_AS), NaAlSiO_4_, and CT. However, for the calcined dealkalization red mud, the cementitious minerals C_4_AF and C_2_S become the main phase of the calcined product (Figure 11b). The results further declare the practicability of the diluted acid leaching strategy in the construction and building industry.

## 4. Conclusions

In summary, an eco-friendly diluted acid leaching strategy was achieved by controlling the phase transformation of red mud during active roasting. The thermal phase transformation behavior of minerals in red mud was studied in detail. Additionally, the phase transformation and related temperature range were determined and analyzed by the TG curve, and three mass loss stages were presented at 200–400, 450–500, and 600–700 °C, respectively. Then, the phase transformation in red mud during the active roasting process was clarified. The results showed that when the calcined temperature was increased to 500 °C, hydrous minerals (FeO(OH) and AlO(OH)) were dehydrated. This was conducive to breaking the connection between red mud particles and loosening the framework structure. When the calcination temperature reached 800 °C, the sodium aluminosilicates (1.08Na_2_O·Al_2_O_3_·1.68SiO_2_·1.8H_2_O) would absorb active SiO_2_ and Al_2_O_3_ and converted into the high Si/Al ratio phases, such as NaAlSiO_4_ and Na_6.8_(Al_6.3_Si_9.7_O_32_). After acid leaching treatment, it was found the 1.08Na_2_O·Al_2_O_3_·1.68SiO_2_·1.8H_2_O could be completely removed by low-concentration acid solution (0.1 M), implying the diluted acid leaching was available with controlling phase transformation. The relevant dealkalization rate was evaluated to be 92.8% of RM-500A. Meanwhile, the leachate was neutral, and the valuable metals could be preserved, such as Fe, Al, and Ti. The results indicated the diluted acid leaching strategy was beneficial to acid saving and valuable metals recovery, without causing secondary pollution. In the calcination experiment, the cementitious minerals C_4_AF and C_2_S became the main phase after calcining RM-500A with limestone, which further confirms the practicability of the strategy in the construction and building field. The diluted acid leaching strategy in this work may promote the development of environmentally friendly dealkalization and red mud resource utilization.

## Figures and Tables

**Table 1:** The chemical composition of raw red mud (wt.%).

Sample	Fe_2_O_3_	Al_2_O_3_	SiO_2_	TiO_2_	Na_2_O	CaO
Raw red mud	30.34	23.59	15.00	6.62	12.44	2.03

**Table 2:** The pH of leachate and the relevant weight loss of leached red mud samples.

Sample	pH of Leachate	Mass Loss/wt.%
RM-800-A0.1	7	21.08
RM-800-A0.25	5	34.67
RM-800-A0.5	1	36.18
RM-800-A1	1	37.97

**Table 3:** The chemical composition, weight loss, and the dealkalization rate of samples (wt.%).

Sample	Fe	Al	Si	Na	Ti	Ca	Mass Loss/%	Dealkalization Rate/%
Raw RM	30.34	23.59	15.00	12.44	6.62	2.03		
RM-500A	33.00	27.38	11.52	1.02	6.72	0.31	12.73	92.8
RM-800A0.1	35.69	24.34	6.08	2.78	7.91	1.01	21.08	82.4

**Table 4:** Comparison of the dealkalization rate and leaching condition between diluted acid leaching and traditional acid or water leaching.

Ref.	Method	Dealkalization Rate/%	Acid Concentration	Leachate
Gong et al., 2020	Acid leaching	97.6		Acidic
Kaya et al., 2018	Acid leaching	95.7	6 M	Acidic
Zeng et al., 2020	Acid leaching	94.16	1.84 M	
Gupta et al., 2019	Acid leaching	31.9	1 M	Neutral
Liu et al., 2017	Water leaching	>90	Three stages of water leaching	
Zhu et al., 2015	Water leaching	82	Four stages of water leaching	Neutral
This work	Diluted acid leaching	92.8	0.1 M	Neutral

## Data Availability

Data sharing is not applicable to this article.
